# Supplementary material for: The burden of antimicrobial resistance among urinary tract isolates of Escherichia coli in the United States in 2017
Source: PLoS One. 2019 Dec 10;14(12):e0220265. doi: 10.1371/journal.pone.0220265 (PMC6903708; doi:10.1371/journal.pone.0220265)
Supplement: Attachment — Submitted filename: Responses to reviewers comments rev.docx [file pone.0220265.s001.docx]

**Response to reviewer comments:**

**Page 5 Bacterial isolates: If this information is available, it would be useful to state what proportion of isolates were from community-acquired infections and what proportion were from hospital-acquired infections; also, from which anatomical sites were these organisms isolated? If this is not possible, state that these are from both settings and/or that sites are unknown, respectively. Reference 20 is not clear regarding this issue.**

***Authors’ response:*** *The isolates that were tested represent both nosocomial and community-acquired infections from both uncomplicated and complicated UTIs. Unfortunately, we do not have access to enough demographic data to be able to separate isolates from community versus hospital-acquired infections. We have added information on anatomical sites with the majority of the organisms being isolated from a urine culture and 132 isolates being isolates from patients with the Foley catheter.*

**Page 5 Susceptibility testing: Add the CLSI M100-S28 in the references**

***Authors’ response:*** *This has been added to the reference list*

**Page 5 Resistance subsets: Consider stating which beta-lactamase genes in addition to blaCTX-M-15 were screened for. Furthermore, further characterisation of a common genotype, blaCTX-M15, could   explain the persistence of these specific isolates in causing infections.**

***Authors’ response:*** *The isolates were screened in silico for narrow and extended spectrum β-lactamases, including carbapenemases. We have also added a comment stating that the usual β -lactamase profiles observed in these studies have been previously published (references added). [Mendes et al., 2019 doi: 10.1093/ofid/ofz004, Castenheira et al., 2019 doi: 10.1128/AAC.00160-19 and Mendes et al., 2019 doi: 10.1089/mdr.2019.0198].*

**Page 7 Susceptibility of all UTI isolates: Use the term "resistance/resistant" with the appropriate % consistently rather than interchanging it with "non-susceptible".**

***Authors’ response:*** *Changes made to substitute the non-susceptible rates with resistance rates*

**Page 7 Prevalence of ESBL phenotypes Figure 2: Consider adding amikacin to figure 2. The high rate of amikacin susceptibility in the ESBL subset provides a carbapenem-sparing treatment option.**

***Authors’ response:*** *Figure 2 has been amended to include amikacin.*

**Page 8 Figure 3: Showing ertapenem resistance rates would yield more useful information than meropenem does. It is clear that all isolates (ESBL and non-ESBL) are meropenem susceptible from Table 1. Ertapenem is a narrower spectrum carbapenem and where possible should be used preferentially.**

***Authors’ response:*** *Yes, we agree but unfortunately not all the 287 ESBL phenotypes of E. coli were tested against ertapenem (only 141 isolates had available susceptibility results for ertapenem) so we selected meropenem as a representative carbapenem since data was available for all 287 ESBL phenotypes for these three drugs.*

**Page 8 Co-resistance among fluoroquinolone-resistant Table 2: Consider adding the amoxicillin-clavulanate data.**

***Authors’ response:*** *Although only 8.6% of the levofloxacin-resistant isolates were resistant to amoxicillin-clavulanate, 34.3% of the isolates displayed were in the intermediate category that would not be reflected in the current presentation of the table and was a factor in our reason not to include.*

**Page 8 Susceptibility of blaCTX-M-15 genotypes: State what % the 151 represents of the total ESBL isolates. If any other predominating beta-lactamase type was found, add this information. If not, state this.**

***Authors’ response:*** *This been added to the text to highlight that CTX-M-15 was the most prevalent accounting for 59% of the ESBL phenotypes of E. coli. Also highlighted that OXA-1/30 was the next most prevalent but in most isolates was co-expressed with CTX-M-15.*

**Page 8 Susceptibility of blaCTX-M-15 genotypes Table 3: Correct year in title of Table 3 (2017 rather than 2016).**

***Authors’ response:*** *This has now been corrected to reflect 2017*

**Page 8 Susceptibility of blaCTX-M-15 genotypes Line 221: Should read "of the carbapenems" not "carbapenem".**

***Authors’ response:*** *This has been corrected*

**Page 9 Paragraph 2: Comment on the % blaCTX-M-15 found.**

***Authors’ response:*** *Added the following text: “Furthermore, bla_CTX-M-15_ was the most prevalent genotype among the UTI isolates of E. coli accounting for 59% of all the ESBL phenotypes*

**Page 10: Nitrofurantoin and fosfomycin data are not presented-add this as a limitation of the study. Authors discuss the need for new oral antibiotics particularly from carbapenem class but perhaps testing of fosfomycin would have shown susceptibility among these pathogens. Fosfomycin is an old antibiotic, used as an oral treatment for uncomplicated urinary tract infections. Fosfomycin has been shown to have activity against some resistant uropathogens suggesting that this antibiotic may provide a useful option for the treatment of patients with difficult-to-treat-infections.**

***Authors’ response:*** *We have added some text to the discussion regarding fosfomycin and nitrofurantoin as oral options for uncomplicated UTI. We included an explanation of why fosfomycin was not tested because of the requirement for agar dilution and therefore a limitation of the current study. Also included a sentence on nitrofurantoin and although a good option of uUTIs caused by E. coli it is not suitable for complicated UTIs where other pathogens such as K. pneumoniae and P. mirabilis are not well covered with this agent.*

**Additional Author Response to Reviewer Questions:**

**1. We noticed you have some minor occurrence(s) of overlapping text with the following previous publication(s), which needs to be addressed:**
[**https://aac.asm.org/content/61/11/e01045-17**](https://aac.asm.org/content/61/11/e01045-17)

**In your revision ensure you cite all your sources (including your own works), and quote or rephrase any duplicated text outside the Methods section. Further consideration is dependent on these concerns being addressed.**

***Authors’ response:*** *All the overlapping text is from the methods section only and there is* ***no*** *duplicated text from outside the Methods section. Both studies were conducted in the same laboratory and used the same methods to molecularly characterize the isolates. To make it clear we have added a reference citation in the methods section to the article by Sader et al*. *(*[*https://aac.asm.org/content/61/11/e01045-17*](https://aac.asm.org/content/61/11/e01045-17)*) to reference the methods used in that study.*

**2. Thank you for including your competing interests statement; "Ian Critchley, Nicole Cotroneo and Michael J. Pucci are employees of Spero Therapeutics. Rodrigo Mendes is an employee of JMI Laboratories"**

**We note that one or more of the authors are employed by a commercial company: name of commercial company.**

***Authors’ response:*** *This does not alter our adherence to PLOS ONE policies on sharing data and materials (as detailed in your guide for authors* [*http://journals.plos.org/plosone/s/competing-interests*](http://journals.plos.org/plosone/s/competing-interests)*).*

3. **Please provide an amended Funding Statement declaring this commercial affiliation, as well as a statement regarding the Role of Funders in your study. If the funding organization did not play a role in the study design, data collection and analysis, decision to publish, or preparation of the manuscript and only provided financial support in the form of authors' salaries and/or research materials, please review your statements relating to the author contributions, and ensure you have specifically and accurately indicated the role(s) that these authors had in your study. You can update author roles in the Author Contributions section of the online submission form.**

***Authors’ response:*** *Our respective employers (Spero Therapeutics and JMI Laboratories) did not have any role in the study design and only provided financial support in the form of salaries and research materials. All authors played an equal role in the design and analyses and presentation of the data arising from this surveillance study.*

Please also include the following statement within your amended Funding Statement.

“The funder provided support in the form of salaries for authors [insert relevant initials], but did not have any additional role in the study design, data collection and analysis, decision to publish, or preparation of the manuscript. The specific roles of these authors are articulated in the ‘author contributions’ section.”

If your commercial affiliation did play a role in your study, please state and explain this role within your updated Funding Statement.

2. Please also provide an updated Competing Interests Statement declaring this commercial affiliation along with any other relevant declarations relating to employment, consultancy, patents, products in development, or marketed products, etc.

Within your Competing Interests Statement, please confirm that this commercial affiliation does not alter your adherence to all PLOS ONE policies on sharing data and materials by including the following statement: "This does not alter our adherence to  PLOS ONE policies on sharing data and materials.” (as detailed online in our guide for authors <http://journals.plos.org/plosone/s/competing-interests>) . If this adherence statement is not accurate and  there are restrictions on sharing of data and/or materials, please state these. Please note that we cannot proceed with consideration of your article until this information has been declared.

***Authors’ response:* AS stated above this does not alter our adherence to PLOS ONE policies on sharing data and materials.**

Please include both an updated Funding Statement and Competing Interests Statement in your cover letter. We will change the online submission form on your behalf.

Please know it is PLOS ONE policy for corresponding authors to declare, on behalf of all authors, all potential competing interests for the purposes of transparency. PLOS defines a competing interest as anything that interferes with, or could reasonably be perceived as interfering with, the full and objective presentation, peer review, editorial decision-making, or publication of research or non-research articles submitted to one of the journals. Competing interests can be financial or non-financial, professional, or personal. Competing interests can arise in relationship to an organization or another person. Please follow this link to our website for more details on competing interests: <http://journals.plos.org/plosone/s/competing-interests>
